# Mixed Actinomycotic-Pyogenic Liver Abscess With Penicillin-Intermediate, Clindamycin-Resistant Actinomyces odontolyticus Bacteremia: A Case Report

**DOI:** 10.7759/cureus.111592

**Published:** 2026-06-27

**Authors:** Aqeel Saleem, Nawal Mohamed, Zaid Al Hassani, Shahd Mohammad, Ali Al Hassani

**Affiliations:** 1 Infectious Diseases, Sheikh Tahnoon Bin Mohammed Medical City, Al Ain, ARE; 2 Internal Medicine, Sheikh Tahnoon Bin Mohammed Medical City, Al Ain, ARE; 3 General Practice, Sheikh Tahnoon Bin Mohammed Medical City, Al Ain, ARE; 4 Clinical Pharmacy, Sheikh Khalifa Medical City, Abu Dhabi, ARE

**Keywords:** actinomyces odontolyticus, antimicrobial resistance, hepatic actinomycosis, liver abscess, streptococcus intermedius

## Abstract

Hepatic actinomycosis is an uncommon diagnostic mimic of pyogenic or amoebic liver abscess. A 34-year-old healthy man presented with fever, vomiting, and right upper quadrant pain after recent travel. Computed tomography showed a 7 x 7 x 5 cm subcapsular segment VIII hepatic abscess. Empirical piperacillin-tazobactam and metronidazole were started, followed by ultrasound-guided drainage with catheter placement. Blood cultures grew *Actinomyces odontolyticus,* and the abscess aspirate grew *Streptococcus intermedius*, supporting a mixed actinomycotic-pyogenic abscess rather than incidental bacteremia. Low-titer *Entamoeba histolytica* serology was not considered clinically significant. The *Actinomyces* isolate showed intermediate susceptibility to penicillin and resistance to clindamycin. After a partial response to amoxicillin-clavulanate, therapy was changed to doxycycline with serial ultrasound surveillance showing interval cavity regression. The case emphasizes contextual interpretation of *Actinomyces* bacteremia, early source control, and susceptibility-guided prolonged therapy.

## Introduction

Actinomycosis is an uncommon invasive infection caused by filamentous Gram-positive bacteria that normally colonize the oropharynx, gastrointestinal tract, and genitourinary tract. It is classically indolent, suppurative, and frequently polymicrobial, which can delay diagnosis because it may mimic malignancy, tuberculosis, or other chronic inflammatory conditions [1,2].

Hepatic actinomycosis is a rare form of abdominal actinomycosis and often overlaps clinically and radiologically with pyogenic liver abscess, amoebic liver abscess, and inflammatory or malignant hepatic lesions [3,4]. *Actinomyces odontolyticus* is an uncommon cause of invasive disease, but bloodstream isolation should be interpreted carefully when a compatible deep-seated infection is present [5,6]. This report highlights a diagnostic and management challenge: mixed actinomycotic-pyogenic hepatic abscess with penicillin-intermediate, clindamycin-resistant *A. odontolyticus* bacteremia.

## Case presentation

A 34-year-old healthy man presented with a one-week history of right upper quadrant abdominal pain and a three-day history of fever, chills, mild headache, and non-bloody vomiting. He had returned from South Asia three months before the presentation. He reported no chronic illness, previous hepatobiliary disease, recent invasive procedure, or previous similar episode.

On admission, he had a temperature of 37.8°C and remained hemodynamically stable. Abdominal examination revealed right upper quadrant tenderness without guarding, rigidity, or peritoneal signs. There was no clinical jaundice or stigmata of chronic liver disease. Initial investigations demonstrated systemic inflammation, hepatocellular transaminitis, and mild hyperbilirubinemia with preserved renal function. The clinical course is summarized in Table 1.

**Table 1:** Clinical course timeline

Time point	Clinical/imaging findings	Management decision
Presentation	Fever, vomiting, and right upper quadrant pain; systemic inflammation and liver enzyme elevation; computed tomography showed a 7 x 7 x 5 cm subcapsular segment VIII hepatic abscess.	Empirical intravenous piperacillin-tazobactam and metronidazole were started.
Day 1	Drainable hepatic collection confirmed.	Ultrasound-guided percutaneous drainage was performed with placement of a 10-French catheter.
Day 5	Repeat computed tomography confirmed appropriate catheter position and reduction in the liquefied component; pleural effusions with right lower-lobe consolidation were also identified.	Antimicrobial therapy was continued, and right-sided pleural drainage was performed.
Discharge and early follow-up	Clinical improvement allowed discharge; later, persistent right upper quadrant discomfort and fatigue prompted review of the susceptibility profile.	Oral amoxicillin-clavulanate was changed to doxycycline 100 mg twice daily.
Six and 12 weeks	The residual hepatic cavity decreased from approximately 4.5 x 3.4 cm to 3.3 x 2.6 cm on ultrasound.	Prolonged therapy and imaging surveillance were continued.

Investigations

Baseline blood tests showed leukocytosis with neutrophilia, markedly elevated C-reactive protein, raised alanine aminotransferase and aspartate aminotransferase levels, mild alkaline phosphatase elevation, and mild hyperbilirubinemia (Table 2).

**Table 2:** Laboratory investigations at admission

Parameter	Result	Reference range
White blood cell count	16.0 x 10^9/L	4.0-11.0 x 10^9/L
Neutrophils	79.6%	40-75%
C-reactive protein	232 mg/L	0-5 mg/L
Alanine aminotransferase	239 U/L	0-41 U/L
Aspartate aminotransferase	172 U/L	0-40 U/L
Alkaline phosphatase	154 U/L	40-129 U/L
Total bilirubin	32.5 micromol/L	3-21 micromol/L
Creatinine	88 micromol/L	62-106 micromol/L

Contrast-enhanced computed tomography of the abdomen showed a 7 x 7 x 5 cm subcapsular segment VIII hepatic abscess with faint peripheral enhancement and no additional intra-abdominal collections or biliary abnormality (Figure 1).

**Figure 1:**
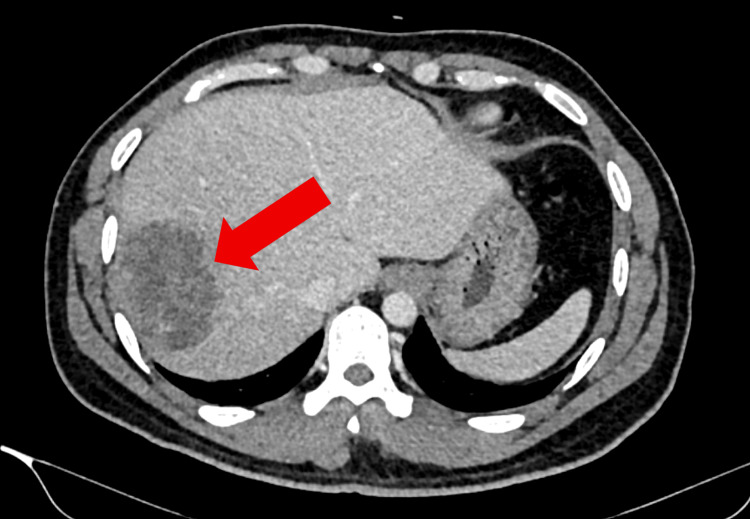
Contrast-enhanced computed tomography of the abdomen demonstrating a subcapsular segment VIII hepatic abscess with faint peripheral enhancement (arrow)

Blood cultures grew *A. odontolyticus*, whereas the abscess aspirate culture yielded *Streptococcus intermedius*. *Actinomyces* are mucosal commensals that can cause invasive infection after mucosal disruption. No clear source was identified in this patient, making an occult oral or gastrointestinal origin possible but ultimately cryptogenic. The absence of *S.* *intermedius *in blood culture did not exclude its role in the hepatic abscess. As a member of the *Streptococcus* *anginosus *group, it is strongly associated with deep abscess formation and may reach the liver through multiple routes. Bloodstream detection may be transient or absent, especially after early antimicrobial therapy, whereas aspirate culture samples the infected cavity directly. Thus, the findings were interpreted as a mixed actinomycotic-pyogenic liver abscess, with *A.* *odontolyticus *bacteremia and *S.* *intermedius *isolated from the abscess cavity. *Entamoeba histolytica *serology was low titer at 1:80 and was not considered clinically significant because culture-proven bacterial infection provided a more direct explanation. The *Actinomyces* isolate later demonstrated intermediate susceptibility to penicillin and resistance to clindamycin (Table 3).

**Table 3:** Microbiological findings and antimicrobial susceptibility

Specimen/test	Organism/result	Susceptibility profile	Clinical interpretation
Blood culture	Actinomyces odontolyticus	Penicillin: intermediate; clindamycin: resistant	Supported clinically significant *Actinomyces *bacteremia in the setting of fever, systemic inflammation, and a compatible deep hepatic abscess. The susceptibility profile reduced confidence in standard penicillin-based therapy alone and excluded clindamycin as a preferred alternative.
Abscess aspirate culture	*Streptococcus intermedius*, heavy growth from enrichment culture only	Ampicillin: susceptible; azithromycin: susceptible; ceftriaxone: susceptible; clindamycin: susceptible; tetracycline: susceptible; vancomycin: susceptible	Confirmed a pyogenic abscess-forming component. Susceptibility to beta-lactams supported the initial inpatient regimen, while tetracycline susceptibility provided additional support for doxycycline as an oral step-down agent when *Actinomyces*-directed therapy was reassessed.
*Entamoeba histolytica* serology	Low titer, 1:80	Not applicable	Not considered clinically significant in the presence of culture-proven bacterial infection.
Antimicrobial interpretation	*Actinomyces*-directed therapy	Metronidazole is not considered a reliable definitive therapy for *Actinomyces* species	Metronidazole was reasonable empirically for possible amoebic or anaerobic liver abscess, but it did not provide dependable *Actinomyces*-directed coverage. Definitive therapy was therefore guided by culture results, susceptibility testing, clinical response, and the need for prolonged oral therapy.

Differential diagnosis

The initial differential diagnosis included pyogenic liver abscess, amoebic liver abscess, and, less likely, malignant or inflammatory hepatic lesions. A pyogenic abscess was supported by fever, systemic inflammation, a drainable hepatic collection, and the subsequent growth of *S.* *intermedius* from the abscess aspirate. Amoebic liver abscess was considered because of recent travel and hepatic abscess morphology, but the low-titer serology and bacterial culture results argued against it as the primary diagnosis [7,8].

Hepatic malignancy or inflammatory pseudotumor remained an imaging consideration at presentation because actinomycosis can mimic these entities [3,4,9]. However, the purulent collection, positive blood and abscess cultures, reduction after drainage, and serial radiological improvement favored infection. The combination of *A. odontolyticus* bacteremia and *S. intermedius* abscess culture supported a mixed actinomycotic-pyogenic abscess rather than contamination.

Treatment

Empirical intravenous piperacillin-tazobactam and metronidazole were started to cover pyogenic liver abscess pathogens, anaerobes, and possible amoebic liver abscess while microbiological confirmation was pending. On day 1, ultrasound-guided percutaneous drainage was performed with placement of a 10-French catheter. Repeat computed tomography on day 5 confirmed appropriate catheter positioning and reduction in the liquefied abscess component (Figure 2A).

**Figure 2:**
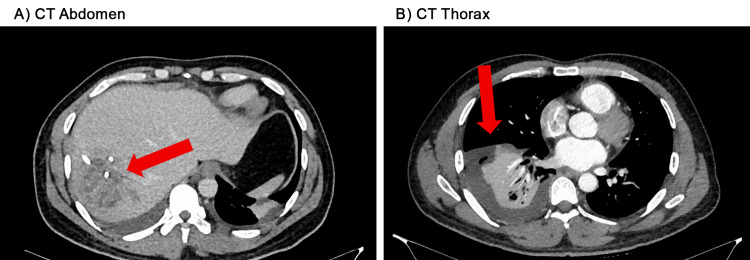
In-admission post-drainage computed tomography findings (A) Day 5 contrast-enhanced computed tomography of the abdomen demonstrating appropriate positioning of the percutaneous hepatic drainage catheter, with interval reduction in the liquefied component of the segment VIII hepatic abscess (arrow). (B) Computed tomography of the thorax performed during the same admission showed bilateral pleural effusions, more prominent on the right, with associated right lower-lobe consolidation (arrow).

During the same admission, computed tomography of the thorax showed bilateral pleural effusions, more prominent on the right, with right lower-lobe consolidation (Figure 2B). Right-sided pleural drainage was performed, and pleural fluid culture showed no bacterial growth. The patient completed inpatient piperacillin-tazobactam and was discharged on oral amoxicillin-clavulanate.

At outpatient review, persistent right upper quadrant discomfort and fatigue, together with the antimicrobial susceptibility profile, prompted reassessment of therapy (Table 3). The *S. intermediu*s abscess isolate was susceptible to ampicillin, azithromycin, ceftriaxone, clindamycin, tetracycline, and vancomycin, supporting that the pyogenic component was broadly susceptible. In contrast, the *A. odontolyticus *blood isolate showed intermediate susceptibility to penicillin and resistance to clindamycin. Metronidazole, although appropriate empirically while amoebic liver abscess and anaerobic pyogenic infection were being considered, is not considered a reliable definitive therapy for *Actinomyces* species. Therefore, because of persistent symptoms, partial response on amoxicillin-clavulanate, reduced confidence in penicillin-based therapy, clindamycin resistance, and the need for a prolonged oral regimen, therapy was changed to doxycycline 100 mg orally twice daily with close clinical and imaging follow-up. This decision was also supported by published actinomycosis literature describing doxycycline as an alternative agent in selected cases when standard beta-lactam therapy is unsuitable or less reliable [1,10].

Outcome and follow-up

Serial ultrasound demonstrated interval regression of the hepatic cavity. At six weeks, the residual cavity measured approximately 4.5 x 3.4 cm. At 12 weeks, the residual right-lobe lesion had decreased to approximately 3.3 x 2.6 cm (Figure 3). These findings were consistent with a favorable interval response to source control and prolonged antimicrobial therapy. Complete radiographic resolution was not yet available at the time of manuscript preparation, so continued follow-up was planned.

**Figure 3:**
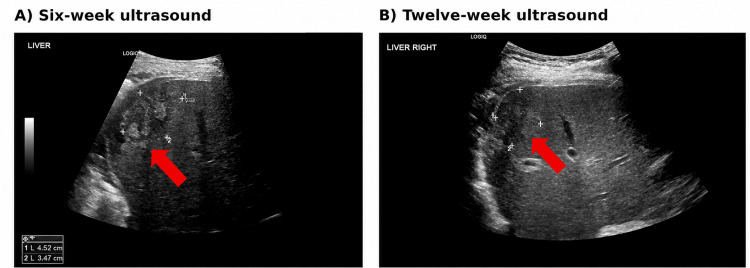
Follow-up ultrasonography demonstrated interval regression of the hepatic abscess cavity (A) At six weeks, the residual cavity measured approximately 4.5 × 3.4 cm (arrow), and (B) at 12 weeks, the right hepatic lobe lesion had decreased to approximately 3.3 × 2.6 cm (arrow).

## Discussion

Case summary and clinical significance

This case is most persuasive when discussed as a mixed actinomycotic-pyogenic hepatic abscess rather than as an isolated monomicrobial hepatic actinomycosis. Several features align with the published hepatic actinomycosis literature: the patient was immunocompetent, the abscess was solitary and right-lobe based, and the presentation was nonspecific enough to overlap with more common liver abscess syndromes [3,4]. At the same time, the species involved makes the case unusual. Published hepatic infections due to *A. odontolyticus *remain sparse. Comparator reports include an immunocompetent patient with *A. odontolyticus *liver abscess, a later HIV-associated mixed hepatic infection in which *A.* *odontolyticus *was recovered along with other organisms, and a 2025 case linking both blood and abscess-fluid growth of *A. odontolyticus *to a perforating gastrointestinal foreign body [11-13]. Against that background, the present case adds an additional clinically relevant feature: a bloodstream isolate interpreted in the setting of a liver abscess plus a susceptibility pattern that made standard penicillin-based expectations less secure.

Pathophysiologic explanation

A central interpretive issue is whether the blood culture isolate should be treated as a true pathogen. In this manuscript, the answer should be yes. Recent data on *Actinomyces* bloodstream infection show that true bacteremia is far more likely when patients have fever, a compatible invasive syndrome, and especially an abscess focus [5,6]. That logic applies directly here: the patient presented with systemic inflammation and imaging-confirmed hepatic suppuration, and there was no more plausible explanation for the bloodstream isolation than the liver abscess itself. Moreover, actinomycosis is often polymicrobial, not purely monomicrobial, because companion microbes can promote tissue invasion and low-oxygen conditions favorable to *Actinomyces* growth [1,2]. The aspirate culture growing *S. intermedius, *therefore, does not weaken the actinomycotic diagnosis; it strengthens the interpretation that this was a polymicrobial deep infection. This is also biologically plausible because *S. intermedius*, a member of the *S. anginosus *group, is strongly associated with deep-seated abscess formation, including liver abscesses [14]. The discussion should therefore state explicitly that the combined finding of *A. odontolyticus *bacteremia plus *S.* *intermedius *in abscess aspirate is more coherent as mixed invasive disease than as contamination plus unrelated pyogenic infection.

Differential diagnosis revisited

The differential diagnosis is also worth sharpening because it illustrates why the case is educational. At presentation, an ordinary pyogenic liver abscess was the most likely working diagnosis and remained justified even after cultures, because the aspirate ultimately confirmed a pyogenic component. The travel history and low-titer *E. histolytica *serology also made amoebic liver abscess a reasonable early consideration. However, the subsequent microbiology shifted the balance away from amoebic disease: bacterial cultures from both blood and abscess provided a more direct etiologic explanation, while low-titer amebic serology alone is insufficiently specific when a patient already has culture-proven bacterial infection [7]. Likewise, hepatic actinomycosis is notorious for mimicking malignant or inflammatory pseudotumor-like lesions, but the drainable purulent lesion, acute inflammatory syndrome, and microbiologic results in this case favor infection over neoplasia [1,3,4,9].

Strengths and limitations of management

For a clinically significant liver abscess of this size, image-guided drainage is standard source control and was a strong decision in this case, both therapeutically and diagnostically. Modern pyogenic liver abscess reviews support drainage when lesions are larger than about 3 cm, loculated, or insufficiently responsive to antibiotics alone [8,15]. That principle dovetails with hepatic actinomycosis literature, in which many reported patients undergo aspiration, drainage, or surgery precisely because diagnosis is uncertain and because tissue or pus is often needed for confirmation [3,9]. Thus, the manuscript should not present drainage as merely a procedural background; it should present drainage as the pivot that converted an undifferentiated liver abscess into a microbiologically characterized mixed infection. Once cultures established the diagnosis, prolonged therapy became the second management pillar. Beta-lactams remain the preferred drugs for most actinomycotic infections, and large hepatic case reviews describe long courses as typical, with a median around four to five months in one 60-year review [1,3,10]. Yet susceptibility data matters. Older and newer susceptibility studies confirm that *Actinomyces* species remain broadly susceptible to beta-lactams and carbapenems, that metronidazole should be avoided as definitive therapy for actinomycosis, and that alternative regimens may be required when resistance or reduced susceptibility is present [10]. In that context, the later switch to doxycycline is reasonable to defend in the discussion: doxycycline is not the canonical first-line drug, but it is an accepted alternative in selected circumstances, especially when standard first-line expectations are undermined by the reported phenotype or by the need for a practical long oral course [1,10].

Several limitations should be acknowledged. First, histopathologic confirmation and sulfur granules were not reported; the diagnosis, therefore, rests on the integration of blood culture, aspirate culture, imaging, and therapeutic response rather than on tissue pathology. Second, the precise portal of entry remains unspecified. Both *Actinomyces* and *S.* *intermedius *are mucosal commensals, so an oral, gastrointestinal, or otherwise disrupted mucosal source is biologically plausible, but no dental, biliary, endoscopic, or colonic source work-up was documented in the supplied materials [1,2,14]. Third, although the patient improved clinically and serial ultrasound showed interval shrinkage of the residual cavity, final radiographic resolution is not yet documented, so the outcome should be described as a favorable interval response rather than a complete cure.

## Conclusions

This case supports interpreting *A.* *odontolyticus *bacteremia as clinically meaningful when it occurs with a compatible hepatic abscess and corroborating microbiologic data. The coexistence of *S.* *intermedius *in the abscess aspirate argues for a mixed actinomycotic-pyogenic process rather than a purely monomicrobial infection. However, the diagnosis was not histopathologically confirmed, as sulfur granules or tissue pathology were not available; therefore, it rests on the integration of blood culture, abscess culture, imaging findings, clinical response, and serial radiologic improvement. The key clinical lessons are diagnostic vigilance, early source control, careful interpretation of blood-culture *Actinomyces* isolates, and prolonged individualized therapy when susceptibility testing departs from standard beta-lactam expectations.
